# Thoracic weighting of restrained subjects during exhaustion recovery causes loss of lung reserve volume in a model of police arrest

**DOI:** 10.1038/s41598-021-94157-w

**Published:** 2021-08-12

**Authors:** Mark Campbell, Roslyn Dakin, Symon Stowe, Kira Burton, Brianna Raven, Malitela Mapani, Jeff W. Dawson, Andy Adler

**Affiliations:** 1 Department of Systems and Computer Engineering, Carleton University, Ottawa, Canada; 2 Department of Biology, Carleton University, Ottawa, Canada

**Keywords:** Biomedical engineering, Experimental models of disease, Respiration, Risk factors

## Abstract

Restraint asphyxia has been proposed as a mechanism for some arrest-related deaths that occur during or shortly after a suspect is taken into custody. Our analysis of the literature found that prone positioning, weight applied to the back, recovery after simulated pursuit, and restraint position have led to restrictive, but non life-threatening respiratory changes when tested in subsets. However, the combined effects of all four parameters have not been tested together in a single study. We hypothesized that a complete protocol with high-sensitivity instrumentation could improve our understanding of breathing physiology during weighted restraint. We designed an electrical impedance tomography (EIT)-based protocol for this purpose and measured the 3D distribution of ventilation within the thorax. Here, we present the results from a study on 17 human subjects that revealed FRC declines during weighted restrained recovery from exercise for subjects in the restraint postures, but not the control posture. These prolonged FRC declines were consistent with abdominal muscle recruitment to assist the inspiratory muscles, suggesting that subjects in restraint postures have increased work of breathing compared to controls. Upon removal of the weighted load, lung reserve volumes gradually increased for the hands-behind-the-head restraint posture but continued to decrease for subjects in the hands-behind-the-back restraint posture. We discuss the possible role this increased work of breathing may play in restraint asphyxia.

## Introduction

Weighted restraint is used in law enforcement, where officers apply their body weight to a subject to control their movements during apprehension. Though rare, arrest-related deaths (ARD) have occurred, and in some cases the cause of death is inexplicable or unknown^1^. Restraint asphyxia has been investigated as a putative mechanism for some of these ARDs^2–8^. Four prominent factors—physical exertion, prone positioning, restraint, and body compression—appear in the statistics of ARDs^9^ and have been tested in other studies, but have not been tested together in a single study. To address this gap in the literature, we have developed a protocol for measuring the combined impacts of these parameters on ventilation using electrical impedance tomography (EIT), which extends our previous work on the topic^10^.

The theory of restraint asphyxia has received criticism in the literature for a lack of evidence supporting it as a mechanism for ARDs^8,11^. The “restriction of thoracic respiratory movements” imparted by a restraint position, together with exertion from pursuit and/or struggle during arrest, has been proposed as a mechanism for inexplicable ARDs^2^. A recent study has found that weighted, prone, restrained positions produced restrictive breathing patterns, causing significant reductions in forced expiratory volume in one second ($\text {FEV}_{1}$) and forced vital capacity (FVC)^6^. Michalewicz et al.^5^ observed that the prone restrained position and the weighted unrestrained prone position each produced independent maximum voluntary ventilation (MVV) decreases in subjects, when comparing measurements taken while subjects were in the seated position. Examples of restraint asphyxia, such as recent highly publicized deaths in police custody, have demonstrated that weighted restraint can lead to restraint asphyxia and that further study of weighted restraint is needed to help inform new police practices.

**Figure 1:**
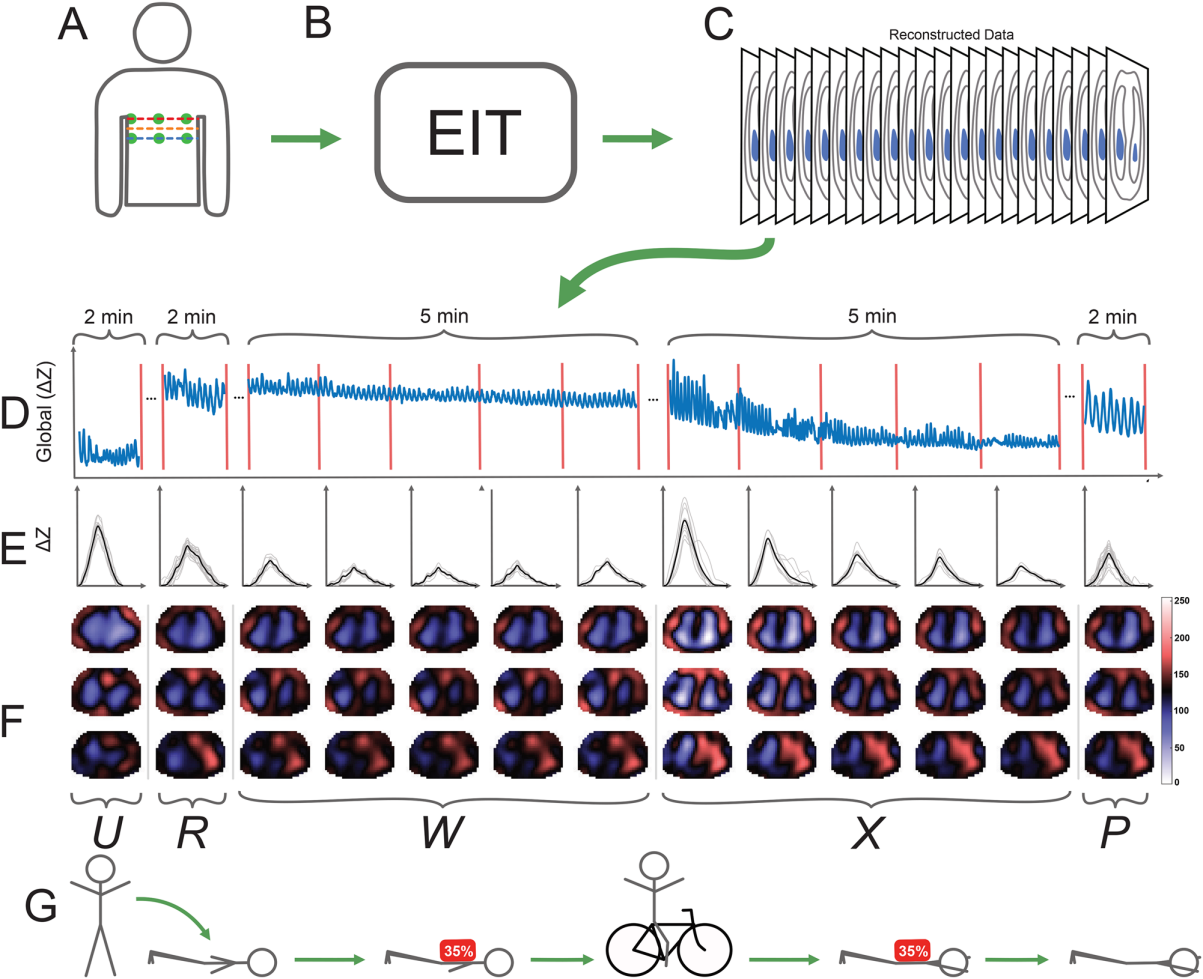
Overview of experimental protocol. (**A**) Two planes of EIT electrodes (2 × 16) were placed on the subject and connected to the SenTec EIT Pioneer Set in a square pattern. (**B**) EIT measurements were recorded for 5 experimental phases: standing upright (U), unweighted prone (R), weighted prone (W), weighted prone in the restraint posture after exercise (X), and unweighted prone in the restraint posture (P). (**C**) Conductivity values reconstructed from voltage data were obtained and used for calculation of parameters. Breath detection was applied on the global sum of $\Delta$Z values of each frame in each phase. (**D**) The global pixel waveform is shown for a sample of each phase (weighted phases were divided into 1-min periods). (**E**) In each phase, breaths were detected (shown as thin lines) from the global $\Delta$Z and the average breath (bold line) was calculated. (**F**) $\text {V}_{T}$ images (see text for details) were reconstructed for 3 planes (show in (**A**)) using the selected breaths shown in (**E**). (**G**) Schematic of experimental phases. The strenuous bicycle exercise task ($\le$10 min) is illustrated between phases W and X. (**A**,**B**,**C**,**G**) were drawn using Inkscape v1.0 (https://inkscape.org). (**D**–**F**) were generated in MATLAB2019a using the EIDORS software package^16^.

EIT is a non-invasive modality which images the distribution of electrical impedance within the body. During inhalation, the low-impedance tissues of the lungs and their surroundings are displaced by high-impedance air, which can measured by EIT in 3D, as shown in Fig. 1A,C,F. These impedance changes during ventilation have been experimentally validated to accurately depict changes in the volume and distribution of air in the lungs^12,13^.

The use of EIT monitoring in weighted restraint makes two important contributions to this field. First, using only body-surface electrodes, EIT allows the research participants to breathe naturally, since no mask or other device must be worn on the face. An EIT-instrumented breathing protocol can therefore be more comfortable and potentially more realistic than other methods. Secondly, functional imaging parameters calculated from reconstructed impedances^14^ have been shown to be more sensitive to changes in lung physiology than global parameters from other methods^15^.

## Results

In this study, we analyzed relative $\dot{V}_{E}$ and relative $\Delta$FRC parameters in subjects while they were standing **upright** (U), in a **reference** prone position with no added weight (R), prone with **weight** (W), prone with weight in one of three restraint postures after an **exercise** task (X), and in the same restraint **posture** as phase X with weight removed (P). CoV was not an important feature for the present study but was included for completeness. Phases U, R, and P were each 2 min long and results were reported as the average for each phase. Phases W and X were each 5 min long, and results were reported as the average for each minute ($\text {W}_{1-5}$ and $\text {X}_{1-5}$).

Each subject was randomly assigned a restraint posture for phases X and P that was one of: (1) a control position of arms at the side (as in R and W), (2) hands clasping each other on the small of the back (hands-back), or (3) both hands on the back of the head (hands-head). Data from 19 subjects were collected. The full protocol as outlined above was completed by 7 subjects (N = 1, 3, 3 for control, hands-back, and hands-head, respectively). The remaining 12 subjects completed all phases except for phase P. The number of subjects that were analyzed by group, for each phase is shown in Table 1.

**Table 1:** Summary of the data that was collected for this study and the data that was analyzed for each treatment group and for each phase.

Group	Number of subjects by phase				
Phase	U	R	W	X	P
Control	5	5	5	5	1
Hands-back	6	6	6	6	3
Hands-head	5	6	6	6	3
Rejected	3	2	2	2	0
Total	19	19	19	19	7

**Figure 2:**
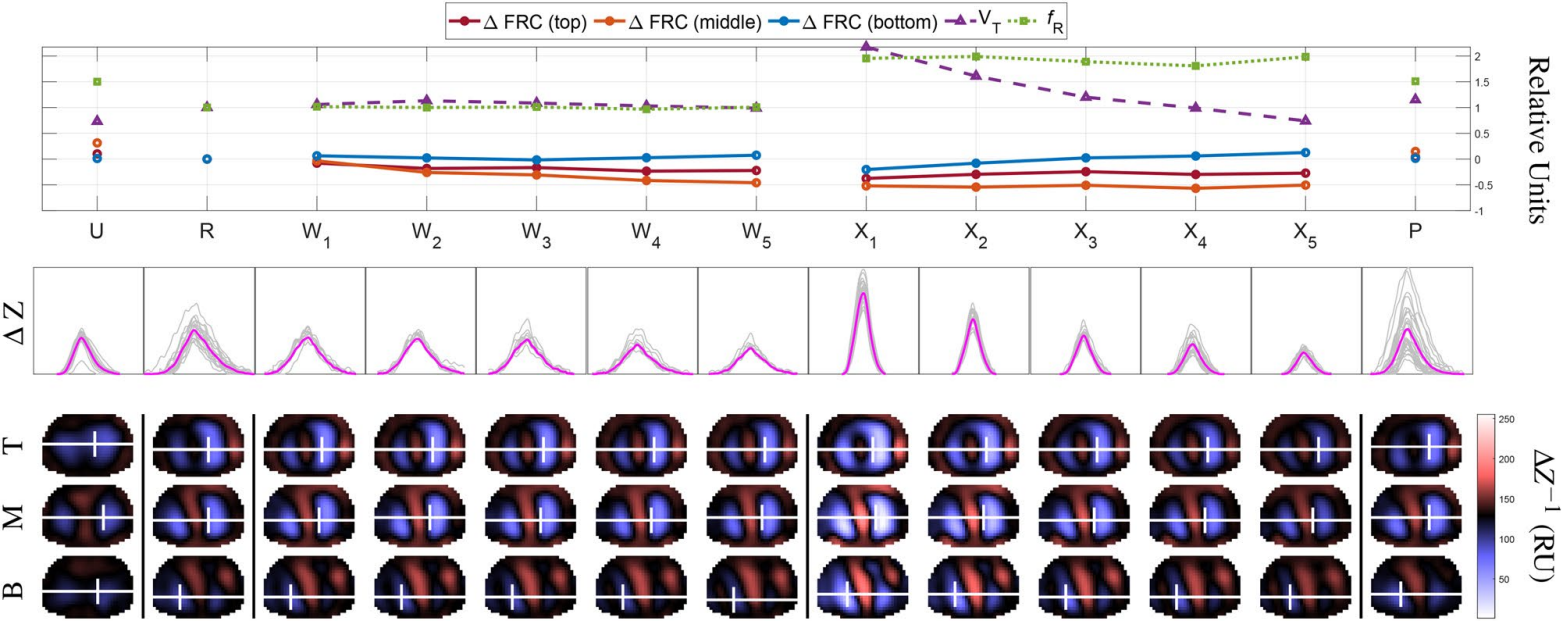
Detailed results for a single subject in the control posture. Experimental phases are labelled as in Fig. 1. Each column in phases W and X were 1-min periods, while phase U, R, and P were a 2-min periods. (Top) plots of $\Delta$FRC the top (red), middle (orange), and bottom (blue) reconstruction planes, $\text {V}_{T}$ (purple), and *f*$_{R}$ (green) for each phase. Error bars were omitted for clarity. Inter-subject variability is shown in Figs. 3 and 4. (Middle) accepted breaths for each phase are plotted in gray along with the average breath in magenta ($\Delta$Z = impedance change from reference frame). All breaths across phases were plotted on the same scale. (Bottom) the internal conductivity distributions for the top (T), middle (M), and bottom (B) reconstruction planes for each phase, with the subject shown face-down. Conductivity (${\Delta }Z^{-1}$) values were scaled across phases, with lower conductivity values corresponding to larger volumes of air in the lungs. The center of ventilation for each reconstruction plane is shown with a white crosshair. The colour bar indicates relative conductivity from low (blue-white) to high (red-white).

Data from phase U for one subject was rejected from analysis due to excessive noise producing ill-defined breath boundaries. The full data from two subjects were also excluded for consistently ill-defined breath boundaries in phases W and/or X. The demographics for the 17 analyzed subjects are summarized in Table 2. The consensus among subjects was that the protocol was uncomfortable but tolerable. All subjects had a GCS score of 15 throughout the protocol. Figure 2 presents detailed observations for a single subject assigned to the control posture.

**Table 2:** Demographics for the full subject pool and for each restraint posture are shown as means ± standard deviations.

Group	Age (years)	Height (cm)	Weight (kg)	BMI (kg m$^{-2}$)
All	25.8 ± 7.86	177 ± 6.15	80.4 ± 14.4	25.7 ± 3.92
Control	22.4 ± 2.56	180 ± 5.58	79.5 ± 10.6	24.4 ± 3.01
Arms-back	24.4 ± 6.07	176 ± 4.16	81.7 ± 15.3	26.2 ± 3.93
Arms-head	30.4 ± 10.4	173 ± 6.32	80.0 ± 16.6	26.4 ± 4.37

BMI: body mass index.

**Figure 3:**
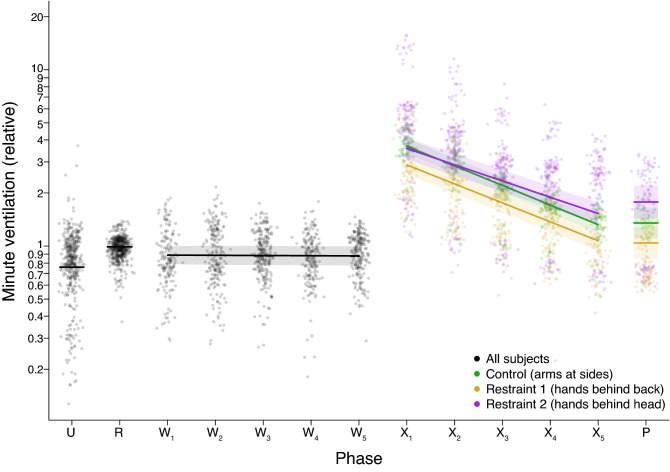
Minute ventilation ($\dot{V}_{E}$), expressed as a ratio of the mean $\dot{V}_{E}$ in phase R, shown on a *ln* scale. Each datapoint represents the $\dot{V}_{E}$ for an individual breath. The solid lines are predicted means derived from the statistical analyses assuming the average age and BMI. The shaded regions show the 95% confidence interval on these model predictions. Postures were either a control posture identical to that of phase W, with arms at the side (N = 5), hands clasping each other on the small of the back (N = 6), or both hands on the back of the head (N = 6). Note that in phase P, the number of subjects was reduced to only N = 1 (control), N = 3 (hands-back) and N = 3 (hands-head).

Our results showed higher $\Delta$FRC values while subjects were standing compared to when they were prone, which indicated that the change in FRC while standing was greater than the FRC change while prone. $\dot{V}_{E}$, shown in Table 3 and Fig. 3, was higher in phase R than phase U.

**Table 3:** Analysis of breathing during initial experimental conditions.

Dependent variable	Comparison	Estimate	SE	z	p-value
$\dot{V}_{E}$ (*ln*-transformed)	R vs. U	0.27	0.03	11.17	< 0.0001
	$\text {W}_{1}$ vs. R	− 0.14	0.03	− 4.58	< 0.0001
$\Delta$FRC	R vs. U	− 0.35	0.01	− 32.67	< 0.0001
	$\text {W}_{1}$ vs. R	0.18	0.02	10.38	< 0.0001

The mixed-effects regression models used to test these contrasts accounted for a subject’s age, their BMI, and the experimental phase in the fixed effects, and included a random effect of subject identity to account for repeated measures (n = 911 breaths from 17 subjects in phases R, U, and $\text {W}_{1}$). To appropriately model the variances, $\dot{V}_{E}$ was *ln*-transformed, and the analysis for $\Delta$FRC accounted for unequal variance across phases. The table provides test statistics for post-hoc contrasts of specific comparisons. U = initial standing upright measurement; R = unweighted prone measurement; $\text {W}_{1}$ = first minute of weighted prone measurement.

**Figure 4:**
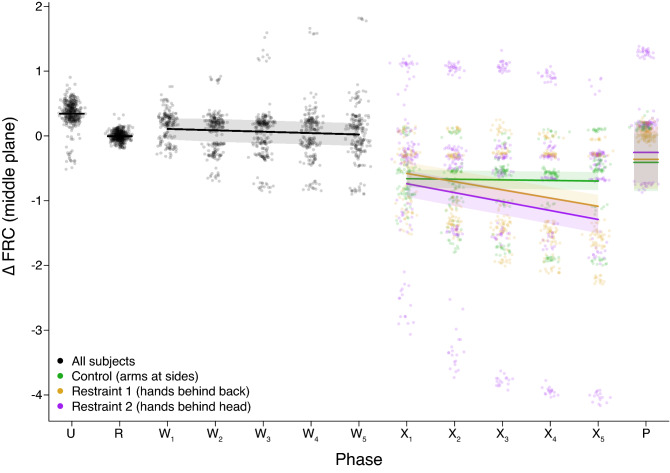
The $\Delta$FRC for each breath in each phase, shown as a relative volume by expressing as a ratio of the mean phase R $\text {V}_{T}$, for the middle imaging plane. The solid lines are predicted means derived from the statistical analyses assuming the average age and BMI. The shaded regions show the 95% confidence interval on these model predictions. Postures were either a control posture identical to that of phase W, with arms at the side (N = 5), hands clasping each other on the small of the back (N = 6), or both hands on the back of the head (N = 6). Note that in phase P, the number of subjects was reduced to only N = 1 (control), N = 3 (hands-back) and N = 3 (hands-head).

We tested whether the addition of weight in phase W would create $\dot{V}_{E}$ and $\Delta$FRC decreases compared to phase R. We observed lower $\dot{V}_{E}$ values when subjects were prone and weighted compared to the unweighted prone measurements, shown in Table 4 and Fig. 3. Contrary to our hypothesis, $\Delta$FRC values (Table 4 and Fig. 4) were positive in $\text {W}_{1}$, then gradually returned to initial phase W values through small but significant decreases for the remainder of 5-min period.

**Table 4:** Analysis of the change in breathing with time during the weighted prone phase W.

Dependent variable	Parameter	Estimate	SE	t	p-value
$\dot{V}_{E}$ (*ln*-transformed)	$\text {b}_{time}$ (change per minute)	0.0007	0.006	0.11	0.91
$\Delta$FRC	$\text {b}_{time}$ (change per minute)	− 0.02	0.0004	− 6.09	< 0.0001

Slope estimates per minute ($\text {b}_{time}$) are provided from mixed-effect regression models that also account for a subject’s age and BMI as fixed effects, and subject identity as a random effect (n = 946 breaths from 17 subjects in the W phase). $\dot{V}_{E}$ is *ln*-transformed.

Next, we tested the hypothesis that restraint postures would be associated with lower $\dot{V}_{E}$ values than the control posture during phase X. The results, presented in Table 6 and Fig. 3, showed $\dot{V}_{E}$ was significantly elevated in phase X following the exercise condition compared to phase W and decreased as subjects recovered. Recovery of $\dot{V}_{E}$ was the slowest for the hands-head posture, being significantly slower than the control and the hands-back postures. No significant differences in $\dot{V}_{E}$ slope were found between the hands-back and control postures.

We predicted that exercise combined with weight and prone restraint during phase X would be associated with loss of FRC, and that the rate of FRC declines would be more dramatic than those observed in phase W. Unlike phase $\text {W}_{1}$, $\Delta$FRC was negative in $\text {X}_{1}$ which represented FRC losses for all postures (Table 5). We observed that $\Delta$FRC decreased more rapidly in phase X than in phase W for the hands-back and hands-head restraint postures, which amounted to losses of 13% and 14% of mean phase R $\text {V}_{T}\,\cdot \,\text{min}^{-1}$ respectively, despite the $\dot{V}_{E}$ decreases that were indicative of exercise recovery. In contrast, the $\Delta$FRC slope for the control posture was near-zero, and we could not reject the null hypothesis that there was no difference between phase W and X slopes for this group (Table 6).

**Table 5:** Analysis of changes in breathing during $\text {X}_{1}$, the first minute of post-exercise weighted restraint (n = 291 breaths from 17 subjects).

Dependent variable	Comparison	Estimate	SE	z	p-value
$\dot{V}_{E}$ (*ln*-transformed)	$\text {X}_{1}$ for control posture	1.35	0.22	6.28	< 0.0001
	$\text {X}_{1}$ for hands-back	1.01	0.19	5.32	0.0002
	$\text {X}_{1}$ for hands-head	1.45	0.20	7.19	< 0.0001
	$\text {X}_{1}$ control vs. $\text {X}_{1}$ hands-back	0.34	0.28	1.20	0.45
	$\text {X}_{1}$ control vs. $\text {X}_{1}$ hands-head	− 0.10	0.31	− 0.33	0.94
	$\text {X}_{1}$ hands-back vs. $\text {X}_{1}$ hands-head	− 0.44	0.29	− 1.54	0.27
$\Delta$FRC	$\text {X}_{1}$ for control posture	− 0.83	0.36	− 2.30	0.04
	$\text {X}_{1}$ for hands-back	− 0.72	0.32	− 2.24	0.045
	$\text {X}_{1}$ for hands-head	− 0.40	0.34	− 1.16	0.27
	$\text {X}_{1}$ control vs. $\text {X}_{1}$ hands-back	− 0.12	0.48	− 0.25	0.97
	$\text {X}_{1}$ control vs. $\text {X}_{1}$ hands-head	− 0.44	0.53	− 0.83	0.69
	$\text {X}_{1}$ hands-back vs. $\text {X}_{1}$ hands-head	− 0.32	0.48	− 0.66	0.79

The first three rows for each parameter test whether the mean of values observed in $\text {X}_{1}$ differed significantly from phase R. $\dot{V}_{E}$ was significantly higher than phase R for all postures and $\Delta$FRC was significantly lower than phase R for the control and hands-back postures, but not the hands-head posture. There were no significant differences between postures for either parameter. Control = posture with arms at side; hands-back = restraint with hands behind back; hands-head = restraint posture with hands behind head.

**Table 6:** Analysis of changes in breathing during post-exercise weighted restraint (phase X).

Dependent variable	Comparison	Estimate	SE	z	p-value
$\dot{V}_{E}$ (*ln*-transformed)	$\text {b}_{time}$ X control vs. $\text {b}_{time}$ W	− 0.25	0.01	− 20.7	< 0.001
	$\text {b}_{time}$ X hands-back vs. $\text {b}_{time}$ W	− 0.24	0.01	− 20.9	< 0.001
	$\text {b}_{time}$ X hands-head vs. $\text {b}_{time}$ W	− 0.21	0.01	− 19.3	< 0.001
	$\text {b}_{time}$ X control vs. $\text {b}_{time}$ X hands-back	− 0.01	0.01	− 0.6	0.98
	$\text {b}_{time}$ X control vs. $\text {b}_{time}$ X hands-head	− 0.04	0.01	− 3.31	0.009
	$\text {b}_{time}$ X hands-back vs. $\text {b}_{time}$ X hands-head	− 0.04	0.01	− 2.78	0.04
$\Delta$FRC	$\text {b}_{time}$ X control vs. $\text {b}_{time}$ W	0.01	0.02	0.77	0.93
	$\text {b}_{time}$ X hands-back vs. $\text {b}_{time}$ W	− 0.11	0.02	− 6.759	< 0.001
	$\text {b}_{time}$ X hands-head vs. $\text {b}_{time}$ W	− 0.12	0.03	− 3.72	0.002
	$\text {b}_{time}$ X control vs. $\text {b}_{time}$ X hands-back	0.12	0.02	5.44	< 0.001
	$\text {b}_{time}$ X control vs. $\text {b}_{time}$ X hands-head	0.13	0.03	3.71	0.002
	$\text {b}_{time}$ X hands-back vs. $\text {b}_{time}$ X hands-head	0.01	0.03	0.33	0.99

The table presents statistical tests of contrasts from mixed-effects regression models where $\text {b}_{time}$ represents slope over time. The analyses account for a subject’s age, BMI, and experimental phase in the fixed effects, and include a random effect of subject identity to account for repeated measures (n = 2441 breaths from 17 subjects in the W and X phases). To appropriately model the variances, $\dot{V}_{E}$ is *ln*-transformed, and the analysis for $\Delta$FRC accounts for unequal variance across treatments. W = weighted prone (prior to exercise); X = weighted prone in the restraint posture (post-exercise); control = posture with arms at side; hands-back = restraint with hands behind back; hands-head = restraint posture with hands behind head.

Finally, we tested whether $\dot{V}_{E}$ and $\Delta$FRC slopes were significantly altered with respect to phase X after removal of the weight in phase P shown in Table 7. $\dot{V}_{E}$ slopes were not significantly different than phase X for the control posture (p = 0.95), were significantly higher for the hands-back posture (p < 0.01), and were higher for the hands-head posture with near significance (p = 0.06). $\dot{V}_{E}$ slopes for the control posture were significantly lower than the hands-back posture (p < 0.01), and lower than the hands-head posture with near significance (p = 0.08) which indicated faster recovery of $\dot{V}_{E}$ for the control posture than the restraint postures. $\Delta$FRC for the control posture showed a small but significant decrease in phase P compared to phase X (p < 0.01). The $\Delta$FRC slope for the hands-back posture was not significantly different between these phases (p = 0.99), but the difference in $\Delta$FRC slope for the hands-head posture was significantly higher in phase P than phase X (p < 0.01). The strength of these findings was limited by the reduced sample size in phase P compared to phase X (Table 1).

**Table 7:** Analysis of recovery in the unweighted prone phase.

Dependent variable	Comparison	Estimate	SE	z	p-value
$\dot{V}_{E}$ (*ln*-transformed)	$\text {b}_{time}$ P vs. $\text {b}_{time}$ X for control	− 0.03	0.06	− 0.52	0.95
	$\text {b}_{time}$ P vs. $\text {b}_{time}$ X for hands-back	0.21	0.03	7.22	< 0.01
	$\text {b}_{time}$ P vs. $\text {b}_{time}$ X for hands-head	0.08	0.03	2.43	0.06
	$\text {b}_{time}$ control vs. $\text {b}_{time}$ hands-back during P	− 0.26	0.07	− 3.88	< 0.01
	$\text {b}_{time}$ control vs. $\text {b}_{time}$ hands-head during P	− 0.16	0.07	− 2.32	0.08
	$\text {b}_{time}$ hands-back vs. $\text {b}_{time}$ hands-head during P	0.10	0.04	2.35	0.08
$\Delta$FRC	$\text {b}_{time}$ P vs. $\text {b}_{time}$ X for control	− 0.07	0.02	− 3.92	< 0.01
	$\text {b}_{time}$ P vs. $\text {b}_{time}$ X for hands-back	0.02	0.11	0.21	0.99
	$\text {b}_{time}$ P vs. $\text {b}_{time}$ X for hands-head	0.25	0.05	4.84	< 0.01
	$\text {b}_{time}$ control vs. $\text {b}_{time}$ hands-back during P	0.06	0.11	0.54	0.95
	$\text {b}_{time}$ control vs. $\text {b}_{time}$ hands-head during P	− 0.22	0.05	− 4.05	< 0.01
	$\text {b}_{time}$ hands-back vs. $\text {b}_{time}$ hands-head during P	− 0.28	0.12	− 2.33	0.07

This analysis compares the slopes ($\text {b}_{time}$) of parameters between the unweighted prone phase, P, and the previous weighted restraint phase, $\text {X}_{1-5}$. Following the removal of the weight, both $\dot{V}_{E}$ and $\Delta$FRC continued to decay with time during the 2 min comprising the unweighted prone phase P. The recovery of $\dot{V}_{E}$ (i.e., its decrease with time) within the P was significantly faster in the control condition as compared to the hands-back treatment. Both restraint postures also slowed the recovery of $\dot{V}_{E}$ in the P phase, as compared to the X phase. $\Delta$FRC continued to decline with time during the P phase. The exception was the hands-head posture, where $\Delta$FRC had a positive slope during the brief P phase. The analyses were mixed-effects regression models that account for a subject’s age, BMI, and experimental phase and treatment in the fixed effects, and include a random effect of subject identity to account for repeated measures (n = 1736 breaths from 17 subjects in the P and X phases). To appropriately model the variances, $\dot{V}_{E}$ is ln-transformed, and the analysis for $\Delta$FRC accounts for unequal variance across treatments.

## Discussion

This study presents a new method for monitoring the combined effects of physical exertion, prone positioning, restraint, and body compression on respiration and ventilation distribution using 3D EIT imaging, shown in Fig. 1. Our main finding was large initial $\Delta$FRC decreases under the combined effects of weight, exercise exhaustion, and prone positioning, which continued to decrease throughout recovery for subjects in restraint postures but not for the control posture, which indicated increased work of breathing for the subjects in the restraint postures. These $\Delta$FRC decreases occurred alongside $\dot{V}_{E}$ recovery rates that were not significantly different between the control and hands-back posture, but were significantly slower for the hands-head posture (Table 6).

The $\Delta$FRC values exhibited large variability between subjects. The analysis controlled for this variability by examining how $\Delta$FRC values changed over time in response to a treatment, which was not influenced by different response magnitudes between phases, at the onset of each treatment. Furthermore, instrumentation errors that may have arisen from postural changes and application/removal of weight were controlled by starting data collection after the treatment had been applied. However, this limited our analysis of $\Delta$FRC in that we could only compare differences in changes of $\Delta$FRC between phases rather than make direct comparisons of $\Delta$FRC.

When subjects transitioned from the standing to prone positions, decreases in $\Delta$FRC were observed, meaning the FRC change in phase U was more positive than the FRC change in phase R. These observations were consistent with previous reports using spirometry that showed FRC was higher while seated than while prone^17^, therefore FRC must increase somewhere in the transition between prone and seated positions, which was seen in our results as a positive $\Delta$FRC for phase U. The $\dot{V}_{E}$ increases observed from the standing position in phase U to the reference prone position in phase R, seen in Fig. 3, were in opposition to the small decreases previously observed between seated and prone positions^17^.

The addition of a 35% bodyweight external static load in phase W was associated with $\Delta$FRC increases, seen in Table 3 and Fig. 4. Given that the physiological response to an inspiratory resistive load involves sympathetic nervous system activation and cardiac acceleration^18,19^, the $\Delta$FRC increases in $\text {W}_{1}$ may have been reflexive hyperinflation of the lungs to counteract the external load. The divergence of $\Delta$FRC values from $\text {W}_{1}$ to $\text {W}_{5}$ seen in Fig. 4 demonstrated that $\Delta$FRC responses to the external load were initially consistent, but responses to the weight over time were heterogeneous, with subject age and BMI accounted for by the mixed-effects regression model used. This heterogeneity of response, specifically subjects with $\Delta$FRC declines in phase W, may be an important factor for predicting the rate of phase X $\Delta$FRC decreases during weighted restraint and exhaustion-recovery. The $\dot{V}_{E}$ decreases observed in phase W were consistent with MVV decreases previously observed between unweighted and weighted prone positions^5^.

The slope of $\dot{V}_{E}$ for each posture group, shown in Table 6 and Fig. 3, was a steeper negative slope than in phase W, as expected during exercise recovery. The shallower $\dot{V}_{E}$ recovery slope for the hands-head restraint posture compared to the hands-back and control postures, with equal relative resistive loads, exercise intensity, and controlling for BMI and age, suggested that this slower recovery rate could be an effect of the hands-head restraint posture itself. The lack of significant difference between $\dot{V}_{E}$ slopes for the control and hands-back posture agreed with a previous study comparing a more restrictive version of the hands-back posture to the control arms-at-the-side posture during recovery from exercise^7^. The hands-head posture was not examined in that study.

The most important observations in this study were the $\Delta$FRC patterns observed in phase X. First, all treatment groups showed relatively large FRC decreases in $\text {X}_{1}$, as shown by the negative $\Delta$FRC values in Fig. 4 and Table 5. These values remained at the same level for the control posture throughout phase X (slope estimate = − 0.01 phase R $\text {V}_{T}$ $\cdot$ min$^{-1}$) while $\dot{V}_{E}$ decreased throughout phase X. A likely reason for this FRC loss was an abdominal muscle recruitment strategy that decreases end-expiratory lung volume to increase the efficiency of the diaphragm when the work of breathing on inspiratory muscles is increased^20^. Secondly, the significant $\Delta$FRC declines seen in phase X for the restraint postures (hands-back slope estimate = − 0.14 phase R $\text {V}_{T}$ $\cdot$ min$^{-1}$, hands-head slope estimate = − 0.13 phase R $\text {V}_{T}$ $\cdot$ min$^{-1}$), but not the control posture (Table 6 and Fig. 4), indicated that the need for abdominal muscle recruitment to assist the diaphragm increased over time for these subjects. Similarly, a pattern of decreasing end-expiratory esophageal pressure, which is indicative of decreasing end-expiratory volume, has been observed with increasing inspiratory resistive loads^21^. Though we did not directly measure muscular effort or the extent of muscular fatigue in phase X, the $\Delta$FRC declines were consistent with increasing inspiratory muscle energy demands over time for subjects in a restraint posture, but not in the control posture. Prolonged increased demand on inspiratory muscles has been shown to impair diaphragmatic function in animal studies^22^, therefore the loss of $\Delta$FRC for subjects in restraint postures suggests they may be more vulnerable to inspiratory muscle fatigue under more extreme conditions.

The difference between $\dot{V}_{E}$ slopes in phase P and X suggested that $\dot{V}_{E}$ recovered faster for the control group than the restraint postures, however the evidence for these trends was weak because data from only one participant in the control posture was collected for phase P (Table 1). The greater $\Delta$FRC slope for the hands-head posture in phase P (slope estimate = 0.12 phase R $\text {V}_{T}$ $\cdot$ min$^{-1}$) compared to phase X was congruent with abdominal muscle decompensation in response to decreased work of breathing after the weight was removed. However, this decompensation did not occur for subjects in the hands-back posture (slope estimate = − 0.12 phase R $\text {V}_{T}$ $\cdot$ min$^{-1}$). Further study quantifying the extent of abdominal and inspiratory muscle fatigue in the restraint posture groups is needed to understand why $\Delta$FRC recovered in the hands-head posture but not the hands-back posture.

The authors recognize certain limitations in our study. First, the imaged lung volume was 6 cm high, compared to the average male lung height of 21 cm^23^. The imaging field measured regions of the lower and middle lobe where the diameter of the lungs is the largest, but information from the apex of the lungs were not captured. Increasing the size of the imaging field in a future study may provide new insights on weighted restraint. Secondly, we placed the weight on the upper back between the scapulae so that the weight would balance during data collection. Applying the weight to the lower back where the ribs are not fused and the thorax is more compliant may have led to more extreme results and is worthy of further study. Third, we could only infer from $\Delta$FRC patterns that work of breathing was higher for the restraint postures than the control group. Collecting EMG data from the abdominal muscles and diaphragm in a future study would allow us to quantify this effect. Finally, our analysis was limited to comparing the changes in $\Delta$FRC between phases to control for the effect of each treatment on the EIT measurements. This experiment could be improved by designing a resting surface and weight container that would not place any pressure on the electrodes so that $\Delta$FRC could be directly compared between phases.

The cardiac effects of weighted restraint are also relevant to the discussion of ARDs. Sudden cardiac death (SCD) is a plausible mechanism for ARDs during weighted restraint, considering that stimulants like cocaine and methamphetamine have been found at autopsy in some cases^8^. Acute restraint stress in a study of rats was associated with increased incidence of SCD from lethal bradycardia and increased cardiac sympathetic activation^24^. Including HR data collection and HR variability analysis in a future study could provide insight on the extent to which different restraint postures contribute to sympathetic nervous system activation.

In summary, our results have shown that subjects in the restraint postures, but not the control posture, experienced continued $\Delta$FRC losses over time during weighted prone recovery from vigorous exercise. These results indicated that the work of breathing in restraint postures was initially the same as the arms-at-the-side control posture, but unlike the control group, gradually increased over time. These $\Delta$FRC losses took place with an applied weight of 35% subject bodyweight, which is likely less than the weight an officer would need to apply to control a suspect, especially if the suspect is violent or uncooperative^5^. Therefore, in true conditions of weighted restraint, we expect that the increasing effort needed to breathe while in a restraint posture would become more relevant to the survival of the subject the longer that the weight is applied. However, further studies using direct measurements of inspiratory muscle fatigue are needed to validate these findings. Finally, we have demonstrated that the ability of EIT to measure changes in respiratory parameters with high sensitivity is well-suited to studies of weighted restraint, where the severity of experimental conditions must be tightly controlled to ensure safety for the research participants.

## Methods

In this study we developed a new 3D-EIT imaging protocol to monitor the combined effects of physical exertion, prone positioning, restraint, and body compression on respiration and ventilation distribution, shown in Fig. 1.

This study took place at Carleton University and was approved by the Carleton University Research Ethics Board, protocol #108481. All experiments were performed according to the guidelines and regulations set out by the Carleton University Research Ethics Board. Nineteen healthy male subjects under the age of 50 (mean: 25.76, range: 19–44) were recruited for this study. All participants provided their written informed consent before taking part in the experiments. Male subjects were selected for this study because they accounted for 95.4% of all ARDs in the United States between 2003 and 2009, according to a 2011 United States Department of Justice technical report^1^. A male sample population was considered representative of the studied population given the 19 times greater prevalence of males in ARDs than females.

Subjects were excluded from this study based on any of the following criteria: a history of anxiety or panic disorders, having exercised for less than 30 min in the last 30 days (considered “not healthy”), being older than 50 years of age, having asthma, answering “yes” to having been diagnosed with a heart or lung condition, feeling unwell, or having a history of back issues.

The age, weight, and height of each subject was first recorded. The subject’s maximum heart rate (MHR) was estimated using the 220-age formula, minus a 10 beat/min safety factor. A total of 32 Ag-AgCl electrodes were placed in a 3D imaging configuration over skin prepared with Nuprep gel, using 2 parallel rings of 16 electrodes arranged in a square pattern. The bottom electrode plane was set 4 cm below the xiphoid process and the top electrode plane was set 6 cm above the bottom plane. This 6 cm tall imaging field captured the widest section of the lungs that included regions of the lower and middle lobes but did not include the apex. EIT measurements were collected using the SenTec EIT Pioneer Set with currents of 3 mA peak-to-peak at 195 kHz in a skip-4 injection and measurement pattern.

The protocol consisted of 5 experimental phases: (1) Standing **upright** for 2 min (U). (2) A **reference** position of prone with arms at the side for 2 min (R). (3) **Weighted** prone positioning with arms at the side for 5 min ($\text {W}_{1}$ − $\text {W}_{5}$). (4) A 5-min weighted prone recovery period after the **exercise** task ($\text {X}_{1}$ − $\text {X}_{5}$) in one of three positions: arms at the side control position (as in R and W), hands clasping each other on the small of the back (hands-back), or both hands on the back of the head (hands-head). (5) Removal of the weight while remaining in the same **posture** as X for 2 min (P). The W and X phases were split into 1-min subphases, denoted by the subscript numbers.

Throughout each recording, subjects’ state of consciousness was monitored using the Glasgow Coma Scale (GCS)^25^ once every 2 min. Recordings with subjects in the prone position took place on a hard foam exercise mat laid over a wooden table, covered with a clean sheet. To breathe comfortably while prone, subjects were given the choice of resting their foreheads on a horseshoe-shaped pillow or turning their heads to the side. In phases W and X, 35% of the subjects’ bodyweight ± 1 kg in bagged sand was placed on the subject’s back, centered over the scapulae where it would not shift or fall during recording. This relative weight was used because it was found to be a good balance between safety for the subject while still being heavy enough to cause discomfort.

Subjects were transferred to a stationary bicycle between phases W and X for the exercise task. This low-impact exercise was selected to minimize the chance of electrode or hardware disconnections. Subjects were given verbal coaching to help them reach the target heart rate (THR) of 70% MHR and maintain it for 3 consecutive min. This THR was chosen because it corresponds to approximately 55% VO$_{2}$ max, placing the subject within the VO$_{2}$ max range needed for cardiorespiratory fitness (CRF) gains^26^. The 3-min exercise duration at the THR was chosen to ensure that participants had been sufficiently exerted, while also ensuring subject safety during the weighted, restrained phase following the exercise task. Exercise was terminated if HR exceeded 85% of their MHR, which would have placed the subject outside of the VO$_{2}$ max range recommended for CRF gains. Otherwise, exercise was terminated if total exercise time exceeded 10 min. All subjects were able to reach and maintain the THR for the required time. Total exercise time ranged from 4 to 6 min. EIT data was not collected during the exercise phase because movement artifacts were expected to make this data unusable.

Subjects were then asked to return to the prone position as quickly as possible and assume their assigned posture. The weight was quickly re-applied, and data was recorded for 5 min ($\text {X}_{1}$ − $\text {X}_{5}$). The weight was immediately removed, then subjects were recorded for another 2 min while maintaining the assigned posture (P). Twelve participants completed Stroop tests during the W and X phases as part of future study examining the cognitive effects of weighted restraint.

The forward model used for solving the internal conductivity distribution was based on the “adult_male” thorax shape in the EIDORS shape library. The lungs were segmented by overlaying the “adult_male_16el_lungs” model over the forward model.

EIT data was reconstructed in MATLAB R2019a using the EIDORS software package^16^. Data was lowpass filtered at 1 Hz using a 57th order Kaiser window FIR filter. The first and last 50 frames of each time series were discarded to remove filter artifacts. Data from 2 participants were excluded due to noise contamination that produced consistently ill-defined breath boundaries.

Breaths were defined by 3 points: the breath inspiration maximum and its two flanking expiration minima. A breath rejection algorithm was used to identify true breaths and reject noise from inadvertent talking or coughing. Parameters were then calculated from the accepted breaths for each phase and sub-phase. Three image planes, one at each electrode ring and one at the midpoint, were reconstructed using the GREIT 3D algorithm^27^.

Changes in pressure on the electrodes was expected to cause voltage shifts between phases, either from the weight of the subjects while prone compared to standing or from the addition of weight. To control for this effect, we started recording each phase after subjects were in the correct position and after the weight had been applied, if applicable. This way, any voltage shifts caused by the treatment would be present throughout each recording and could be cancelled out during image reconstruction. Impedance values for each phase were reconstructed relative to the mean measurement frame of the first accepted breath in each phase. However, this strategy limited our analysis to comparisons of changes in reconstructed impedances between phases, rather than comparisons of reconstructed impedances themselves.

Tidal volume ($\text {V}_{T}$) values were calculated from reconstructed images. For each accepted breath, a $\text {V}_{T}$ image was produced from the impedance difference ($\Delta$Z) observed between its inspiration maximum and the mean of its two flanking expiration minima. Since the $\text {V}_{T}$ images were the difference between two reconstructed images, $\text {V}_{T}$ images could be compared between phases. Each $\text {V}_{T}$ value was taken as the sum of the $\Delta$Z for all pixels within the lung segmentation of a single $\text {V}_{T}$ image. The reconstructed images for an individual subject (as in Fig. 2) show the mean conductivity value ($\Delta$Z$^{-1}$) for each pixel across $\text {V}_{T}$ images for a given timeframe.

Instantaneous respiratory rate (*f*$_{R}$) in breaths $\cdot$ min$^{-1}$ was calculated for each breath using the inverse length of time between expiration minima. *f*$_{R}$ was expressed as the average value of breaths occurring during that phase or sub-phase, then expressed as a ratio of *f*$_{R}$ in phase R. The reconstructed images for phase R were averaged into a single image. In each of the W and X phases, 5 images were produced as the average of accepted breaths occurring in 5 non-overlapping 60 s windows.

We measured relative changes in functional residual capacity ($\Delta$FRC) by first calculating the sum of impedances for all pixels in the lung segmentation at each end-expiration (EELI$_{1}$ and EELI$_{2}$). The mean EELI was then calculated for each accepted breath (Eq. 1).

1 $$\begin{aligned} FRC = \frac{(EELI_{1} + EELI_{2})}{2} \end{aligned}$$

Next, we subtracted the average phase R FRC value from the FRC value of all breaths (Eq. 2), giving $\delta$FRC values. Positive $\delta$FRC values indicate FRC increases relative to phase R and negative $\delta$FRC values indicate FRC decreases relative to phase R. In other words, FRC changes were zeroed to the FRC changes in phase R.

2 $$\begin{aligned} {\delta }FRC_{phase, n} = FRC_{phase, n} - \frac{\sum _{i=1}^n FRC_{phase R, i} }{ n } \end{aligned}$$

Finally, $\delta$FRC values were converted from units of $\Delta$Z to a relative volume by dividing by the mean $\text {V}_{T}$ of phase R (Eq. 3). The $\Delta$FRC units of are V${_{T, phase R}}^{-1}$. This conversion was done so that the $\Delta$FRC values in this study by EIT would be comparable to $\Delta$FRC values obtained by spirometry, where the $\text {V}_{T}$ and FRC measurements would be in units of ml instead of $\Delta$Z. For example, a $\Delta$FRC value of 1, assuming an individual’s mean phase R $\text {V}_{T}$ was 400 ml, would represent an FRC increase of 400 ml. Additionally, this conversion provided a clearer and more physiologically meaningful presentation of the results.

3 $$\begin{aligned} {\Delta }FRC_{phase, n} = \frac{ {\delta }FRC_{phase, n} }{ \frac{ \sum _{i=1}^n V_{T, phase R, i} }{ n } } \end{aligned}$$

$\Delta$FRC values reported for a specific phase show the change in EELI for a given breath relative to the beginning of that phase, which represents the difference between end-expiratory lung volumes between the two time points. Changes in $\Delta$FRC are first order and represent the change in FRC over time.

Minute ventilation ($\dot{V}_{E}$) was calculated as the product of measured *f*$_{R}$ and $\text {V}_{T}$ values, then expressed as a ratio of $\dot{V}_{E}$ at phase R. $\dot{V}_{E}$ was normalized to phase R so that parameter values in other phases could be compared to a common reference point and to control for individual differences in resting $\dot{V}_{E}$. Center of ventilation (CoV) values were calculated as previously described^28^ and are shown as white crosshairs in Fig. 2.

Statistical analyses were conducted using R version 3.6.3^29^. Mixed-effects regression models were fit in the nlme package version 3.1-144, with subject ID as a random effect. All models included age and BMI as fixed effects. Post-hoc comparisons were derived from the multcomp package version 1.4-14 to test specific hypotheses, using the mcp function to derive inferences that account for multiple comparisons. The estimates reported for the comparison between two phases (e.g. R vs. U) are the difference between mean phase parameter values for the two phases. The estimates for parameters that include $\text {b}_{time}$ in the parameter name report the slope of the linear regression fit to the observations for a given phase. The estimates for comparisons of slopes between phases show the difference between the estimated slope for each phase. For example, the $\text {b}_{time}$ X control vs. $\text {b}_{time}$ W estimate is the difference the parameter’s slope in phase X for the control group and the parameter’s slope in phase W. In table Table 7, $\text {b}_{time}$ for phase P was obtained from the slope of parameter averages calculated for the first and second minutes of the phase.

## Data Availability

The datasets generated and analysed during the current study are available in the Scholars Portal Dataverse, found at 10.5683/SP2/WXI8NV.
